# Unveiling the effects of consumers' psychological distance on their reactance and related behavioral outcomes: Do lockdown restrictions matter?

**DOI:** 10.3389/fpsyg.2022.952964

**Published:** 2022-10-03

**Authors:** Xianglan Chen, Yachao Duan, Huma Ittefaq, Yahui Duan

**Affiliations:** ^1^Center for Cognitive Science of Language, Beijing Language and Culture University, Beijing, China; ^2^China Renaissance, Beijing, China; ^3^Institute of Business and Management, University of Engineering and Technology, Lahore, Pakistan; ^4^School of Foreign Language and Application, The Australian National University, Canberra, ACT, Australia

**Keywords:** psychological reactance, consumer reactance, COVID-19, food and beverage restaurants, distancing, compliance

## Abstract

This study examined consumers' psychological reactance, which is insufficiently explored in the literature. This research fills the gaps found in the literature reviewed and investigates how the psychological reactance of restaurant consumers developed because of social, temporal, and spatial distancing measures during COVID-19. This study also explored ways in which the restaurant industry can increase its compliance with COVID-19 restrictions in such a situation. We explored the effects of social, temporal, and spatial distancing on psychological reactance. We also identified the moderating effect of lockdown restrictions, which strengthen the psychological reactance developed because of (a) social distancing, (b) temporal distancing, and (c) spatial distancing. We collected data from restaurant consumers in Lahore. This study applied quantitative techniques (i.e., a test of normality, measurement model assessment, structural model assessment, and common method variance). The data were analyzed using IBM SPSS 25 and AMOS 24 and the results were interpreted and presented accordingly. This study added to the literature on psychological reactance, service management, and psychological distance. We could also help the restaurant industry overcome the challenges that occurred during the COVID-19 pandemic and the closures. This study could assist the restaurant industry to understand consumer behavior and attract potential consumers.

## Introduction

The COVID-19 virus first appeared in the Wuhan city of China in 2019 and then spread to more than 200 countries, becoming a global threat as it caused thousands of deaths globally (Mirza et al., 2020). To avoid the deadly consequences of this pandemic, governments opted to follow WHO guidelines, which are expected to save the lives of at least 20 million people (Van Rooij et al., 2020). These preventive measures include keeping a 2 m distance between each other in public places and other restrictions on social interaction (Abel and Mcqueen, 2020). The COVID-19 pandemic preventative measures include staying at home, closing borders, social isolation, hygiene, wearing masks, and being tested when individuals feel unwell (Sargeant et al., 2021). This outbreak has influenced the world economy by affecting production, financial activities, supply chains of firms, markets, and consumers (Javed, 2020). Therefore, governments imposed restrictions on business activities, such as closures and operating hours to slow their spread, resulting in consumers' psychological reactance (Lakshmi and Shareena, 2020). For instance, restaurants have even paid their salaries and expenditures without earning anything, causing the industry to struggle severely (Lakshmi and Shareena, 2020). Despite the fact that eating habits are gradually improving, making the restaurant business highly competitive, during COVID-19 the restaurant industry is being destroyed due to social, temporal, and spatial distance (Rahoo and Khan, 2021). Because of distance restrictions due to COVID-19, 14–30% of restaurants have been permanently closed, and customers are psychologically uncomfortable. It can be argued that COVID-19 distance limits have an impact on the restaurant industry and are more likely to cause psychological distance and associated responses in consumers.

A stream of literature has focused on COVID-19 and consumer behavioral responses. The government's restrictions and preventive measures during the COVID-19 pandemic made people feel threatened, causing psychological reactance and the restoration of freedom (Hajek and Häfner, 2021). The psychological reactance theory states that when an individual faces any persuasion to change their current behavior or possessed attitude, they may perceive it as a threat to their perceived freedom of choice (Brehm and Cole, 1966). Reactance is a result of the discomfort and anger caused by the restrictions on freedom of movement and interaction (Kokkoris, 2020). When an individual tries to restore freedom, the reactance may result in positive responses or negative responses (Moore and Fitzsimons, 2014). People positively respond to the COVID-19 restrictions and comply with the restrictions imposed by the government only if they understand the concern and have trust in the government (Lalot et al., 2020). We contend that COVID-19 restrictions, such as social, temporal, and spatial distance cause anger and unpleasant feelings in restaurant consumers, resulting in psychological reactance. The restaurant's customers then attempt to restore their perceived freedom.

Clee et al. (1980) stated that consumer psychological reactance extends beyond the existing scope of literature, which covers price, availability, and promotions. Therefore, during COVID-19, we investigated customer psychological reactance because of psychological distance. A few studies referred to the relationship between psychological distancing and psychological reactance (Abel and Mcqueen, 2020; Reiss et al., 2020; Chishima et al., 2021; Finsterwalder, 2021; Zhang et al., 2021) and the psychological reactance to freedom restoration in the study of Moore and Fitzsimons (2014), Bessarabova et al. (2017), and Zhang (2020). The work of Akhtar et al. (2020) and Sakai et al. (2021) also supported the relationship of psychological reactance with COVID-19 restrictions. However, the existing literature has largely ignored the investigation of psychological distancing as a predictor of psychological reactance in freedom restoration and compliance with COVID-19 restrictions. Further, when COVID-19 restrictions cause anger and negative thoughts, people are more likely to follow them. There isn't enough research on how psychological reactance affects compliance with COVID-19 restrictions. Lockdown restrictions have also been shown to improve psychological distancing measures that lead to psychological reactance (Foroudi et al., 2021), but have not been thoroughly investigated as a moderator for the relationship between psychological distancing and psychological reactance. Following that, we filled the gaps by investigating the psychological reactance caused by perceived social, temporal, and spatial distance in restaurants. We used a novel approach in examining consumers' psychological reactance from a psychological distance to restaurant consumers and developed and validated a research framework. We aim to confirm the structural relationships between constructs. First, we examined the consumers' psychological reactance developed by perceived social, temporal, and spatial distance at the food and beverage restaurants. Second, we investigated the freedom restoration and compliance with COVID-19 restrictions as the outcomes of consumers' psychological reactance. Third, we checked the moderation effect of lockdown restrictions reinforces the relationship of social, spatial, and temporal distancing with psychological reactance in the context of the COVID-19 pandemic. We had the objectives for this study to help food and beverage restaurants with understanding the consumers' reactance during the COVID-19 pandemic when the restaurants apply distance measures and how this reactance can affect the much-needed compliance to COVID-19 measures. This study gives an insight into the food and beverage restaurants regarding the consumers' accepted dining, creative ways of applying COVID-19 precautionary measures, and compliance with government restrictions at the same time. We added to the body of knowledge on psychological distance, consumer psychology, service management, and consumer reactance. We also provided implications for the food and beverage industry to help restore consumer freedom.

## Theoretical background and hypotheses development

### Psychological reactance theory

In this study, we used the psychological reactance theory (PRT), which was developed by Brehm in the 1960s (1996). It is defined as when an individual feels that his or her freedom is being constrained by someone, a state of discomfort and unpleasant motivation develops, motivating them to restore their threatened or lost freedom (Miron and Brehm, 2006). This theory has been utilized in various disciplines in the existing research. For example, customer response to product characteristics, such as unavailability and price; political cause endorsement; censorship; consuming styles that have an environmental impact; altruism and helping behavior; and reference groups influence customer behavior (Clee et al., 1980). The degree of psychological reactance is determined by the significance of the threatened freedom and the perceived severity of the threat (Steindl et al., 2015). This study argues that psychological reactions to something that restricts an individual's freedom of choice at a restaurant cause the individual to feel irritated, and they attempt to restore their freedom through behavioral actions.

**Figure 1:**
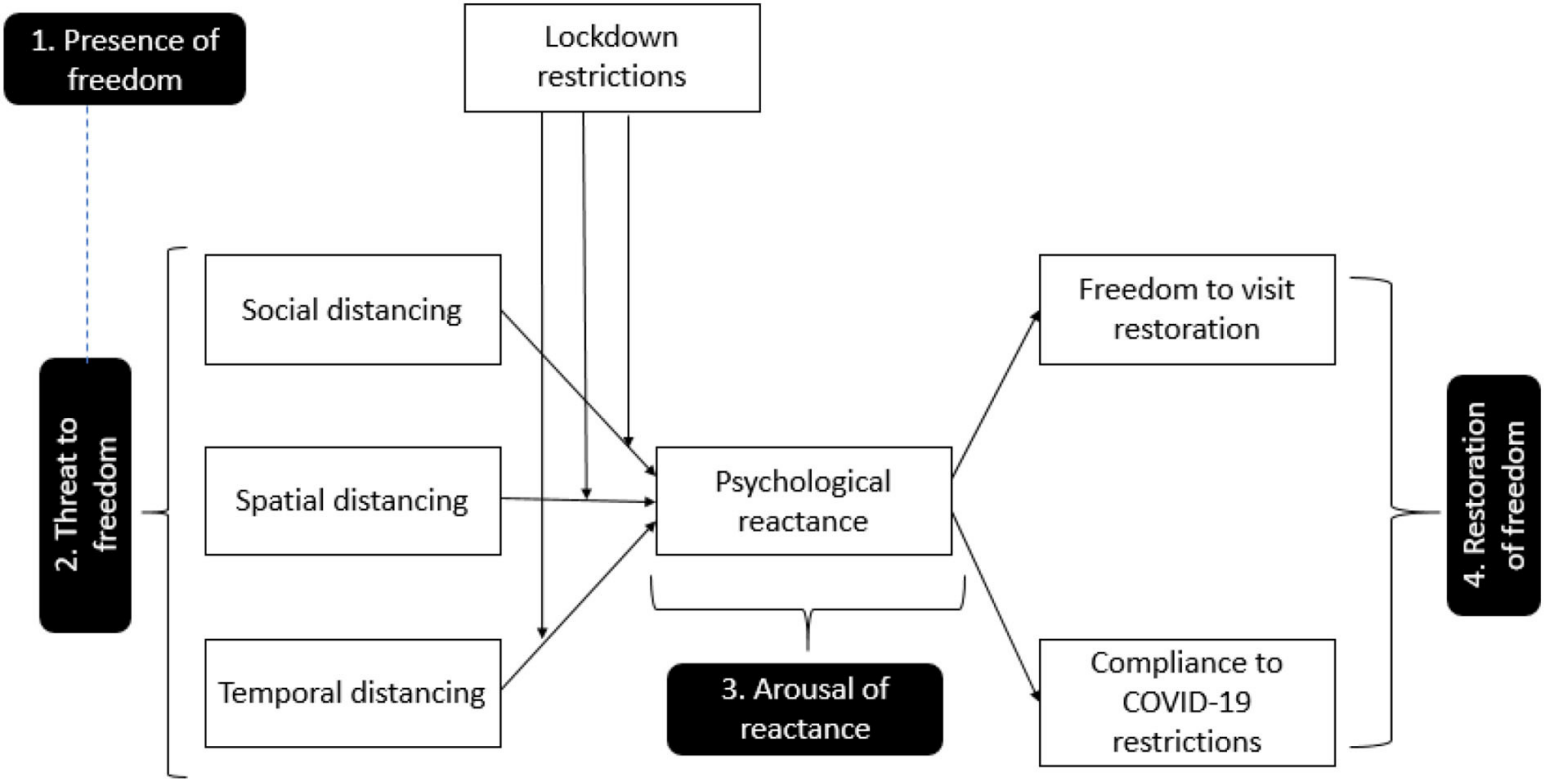
Psychological reactance theory.

Psychological reactance theory has four components: (i) presence of freedom, (ii) elimination of freedom or threat to freedom, (iii) reactance arousal, and (iv) restoration of freedom (Shen and Dillard, 2005). These components in reference to our research model are presented in Figure 1. The aim of the research is to use the four components of psychological reactance to determine restaurant customers' reactions to psychological distancing during the COVID-19 pandemic. First, PRT states that individuals have a set of free behaviors that they can engage in at any time. In the current study context, we assume that restaurant consumers have the freedom to move, sit, interact, and visit the restaurants according to their willingness.

Second, PRT refers to the perceived threat to freedom. Brehm discovered that restrictions on freedom are uncomfortable and that such restrictions, which can be internal or external, motivate people to restore their lost freedom. It can be contended that restaurant customers perceive psychological distance (i.e., social, temporal, and spatial) and lockdown restrictions as a threat to their freedom to move and interact in restaurants. Third, PRT considers arousal of reactance, which refers to unpleasant reactions to threats. The magnitude of the reactance is determined by the significance of the threatened freedom and the severity of the threat. We contend that the threat of psychological distance induces psychological reactance in restaurant customers, which influences their intention to visit and induces preventative behavior. Lastly, this theory relates to the restoration of formerly limited freedom. In this study, we believe that psychological distance and lockdown limits make restaurant customers feel anxious and that they are eager to restore their freedom of movement and visit the restaurants.

Based on the literature, we established the logical connection between the PRT and the study framework. The link between epidemics, such as SARS, Influenza, Ebola, and Swine Flu, has been extensively studied in the literature (Akhtar et al., 2020), demonstrating the relevance of PRT in the context of the COVID-19 pandemic. The primary components of the PRT include the consumers' freedom restriction resulting in anger and negative cognitions (Ding et al., 2021), which provides a foundation for investigating the restaurant consumers' reactions to psychological distance during the COVID-19 pandemic. Prior literature (Rosenberg and Siegel, 2018; Hoang, 2020; Lee et al., 2020; de Rosa and Mannarini, 2021; Fischetti et al., 2021; Foroudi et al., 2021) also lends support to the application of this theory in the current study.

### Hypotheses development

#### Social distancing and psychological reactance

Social distancing means keeping distance from others, and it includes the avoidance of any contact with other people and other groups (Ali et al., 2021). To minimize the spread of the COVID-19 virus, people should keep a 6-foot distance from anyone who is not from their family or home, and wear masks and gloves when going out until they build immunity to the virus (Labe et al., 2021). Social distancing and other mobility restrictions are perceived as losses of freedom and create psychological reactance in people, which motivates them to restore their freedom (Sakai et al., 2021). We argue that consumers take social distancing as a threat to their social freedom of interaction and that when it is used in a restaurant context, it creates psychological reactance, which makes them angry and uncomfortable.

Since COVID-19 is proved to be a threat to humanity, social distancing measures would be helpful as preventive measures against the virus (Labe et al., 2021), but results show that people have different reactions toward compliance with social distancing. The study by Lemenager et al. (2021) shows that COVID-19 lockdown restrictions, including social distancing, create depression and negative emotions among the people and also affect the consumption of social media during the pandemic. Ali et al. (2021) explore the consumer's attitude toward social distancing in relation to their reactions to changes in hospitality brands. The work of DeFranza et al. (2021), predicts low compliance with social distancing as a reactance to restrictions on personal and religious freedom. The above-mentioned literature elucidates the relationship between social distancing and psychological reactance. Thus, we contend that COVID-19 social distance restrictions in food and beverage restaurants limit people's social freedom (to engage with others) and cause reactance, eliciting negative emotions and motivating them to restore their perceived freedom. Hence, we propose the following hypothesis:

**H1:** Social distancing is positively related to the consumers' psychological reactance.

#### Spatial distancing and psychological reactance

Spatial distance means the concept of here vs. there, which basically refers to the perceived distance from the final destination of the object (Cui et al., 2020). According to a study by So et al. (2019), social psychology defines spatial distance as the gap between one's current location and different locations. Yang and McAllister (2020) found that people feel anger, anxiety, fear, and sadness when they are far away from something that makes them think there is a threat to their freedom. The spatial distance creates individual psychological reactance that causes threats to the freedom of in-group people for any wrongdoing, which encourages them to restore their freedom by defending or reacting (Rinck and Bower, 2000). We argue that spatial distancing causes psychological reactance in food and beverage restaurant customers when they feel that the restaurant is physically more distant.

The people who work in the travel and hospitality industries are the worst hit by the disease COVID-19 because it has spread across the world, causing psychological reactance (Lakshmi and Shareena, 2020). Spatial distancing may be a more effective approach in mitigating the negative effects of the COVID-19 pandemic on people's mental health, which includes depression, anxiety, and psychological reactance (Abel and Mcqueen, 2020). Likewise, Yang and Mcallister (2020) show the results of a relationship between spatial distancing and discrete emotions like anger, anxiety, fear, and sadness that cause customer psychological reactance. Furthermore, Holodova (2021) examined the impact of perceived threat on spatial distancing with the mediating role of psychological reactance and shows the relation between spatial distancing and psychological reactance. From the above-mentioned literature, we propose that spatial distancing is perceived as the freedom to threaten by food and beverage restaurant customers and that it creates anger and other negative emotions that result in psychological reactance among the customers. Hence, the following hypothesis is put forward:

**H2:** Spatial distancing is positively related to consumers' psychological reactance.

#### Temporal distancing and psychological reactance

Temporal distance refers to the distance in time which can be between objects, subjects, or events and that can be located at any point of time, near or far away in time, in the future, or in the past (Finsterwalder, 2021). Liberman et al. (2002) define temporal distance as the time difference between the direct experience of a perceiver and a stimulus, which can be an object or event. The research study of Liu et al. (2020) has elaborated that the COVID-19 pandemic has resulted in an increase in temporal distance and an increase in the threat perceived to be near. We contend that temporal distancing includes time constraints and fosters negative emotions and perceptions of threat to freedom, leading to psychological reactance in the context of restaurants.

One of the dimensions of psychological distancing includes temporal distancing, which causes consumer reactance that helps in mitigating the adverse effects of COVID-19 (Li et al., 2020). The study by Chishima et al. (2021) explored temporal distancing in the COVID-19 context and explored consumer negative emotions that develop psychological reactance to restore freedom. The research of Li et al. (2021) examined temporal distancing in the context of restaurants and explored its relationship with consumer evaluation, causing an unpleasant state of motivation. Chung and Park (2017) investigated the psychological distance, including temporal distance, that develops consumers' reactance to restore liberty. From the literature studied, we infer that temporal distancing restricts the freedom of time in the customers of restaurants during COVID-19, which makes them feel uncomfortable and creates psychological reactance to restore the freedom threatened or lost. Hence, we formulate this hypothesis:

**H3:** Temporal distancing is positively related to consumers' psychological reactance.

#### Consumer's psychological reactance and freedom to visit restoration

The restoration of freedom threatened is always accompanied by an increase in psychological reactance, which indicates that the person who is experiencing psychological reactance will want to restore the freedom that has been lost (Clee et al., 1980). People identify the threatened freedom as more valuable after the perceived threat and try to restore it (Kim et al., 2013). A person's desire to restore lost or threatened freedom can be stoked by a variety of tactics and approaches, but the most common way to do so is to oppose persuasion by expressing negative feelings or by doing the opposite (Bessarabova et al., 2017). We argue that after the arousal of psychological reactance because of COVID-19 restrictions on psychological distancing in restaurants, individuals will develop psychological reactance and, thus, they will restore the freedom to visit restaurants.

Heilman and Toffler (1976) aimed at exploring the restoration of freedom in female students when they are offered something vs. when their freedom of choice is threatened. Study Schwarz (1984) explains that a threat can be perceived as more threatening to freedom when it is really not, and an individual can immediately try to restore the threatened freedom. Similarly, Zhang (2020) declares that psychological reactance has a direct relationship with reactance and will result in a negative appraisal of the source of the psychological reactance and that the individual will then try to restore the freedom that was taken away from him. Considering these arguments, we can conclude that psychological reactance will occur during COVID-19 when customer visits are restricted, and psychological distancing is used in restaurants. Restaurant customers will feel discomfort and negative emotions upon feeling the threat to their perceived freedom to visit restaurants according to their will, which then restores the freedom to visit restaurants. Thus, the following hypothesis is developed:

**H4:** Consumer's psychological reactance has a positive relationship with their freedom to visit restoration.

#### Consumer's psychological reactance and compliance to COVID-19 restrictions

Every serious alert to humanity requires everyone to follow some rules and regulations and to follow some restrictions by changing their behavior and the COVID-19 pandemic demands officially recommended precautions like self-quarantining or avoiding social contact therefore compliance from the individuals is very important in such situation, but compliance with these recommendations is a wide topic which retains controversy also (Díaz and Cova, 2021). At the start of the pandemic, it is observed that there was a high degree of compliance with COVID-19 regulations and people use to encourage others to comply with the COVID-19 regulations but with the decrease in the number of infectious people, compliance also decreased (Hajek and Häfner, 2021). The study by Frey et al. (2021), explains that psychological reactance has an impact on the intention of compliance and it mediated the relationship between the threat to freedom and compliance. There is reactance arousal for the COVID-19 restrictions among the individuals which is mainly because of the anger emotion developed in such situations (Hajek and Häfner, 2021). We argue that the COVID-19 restrictions develop psychological reactance among individuals and this psychological reactance affects compliance with COVID-19 restrictions.

Psychological reactance plays an important role in compliance with the persuasive messaging regarding COVID-19 restrictions (Adiwena et al., 2020). With the announcement of the implementation of COVID-19 precautionary measures, people start developing emotions of anger which leads to psychological reactance and affects the intention of compliance (Krpan and Dolan, 2021). The research of Hajek and Häfner (2021), explains that reactance elements tend to have an impact on compliance with COVID-19 restrictions. The research of Sobol et al. (2020), elaborates that the announcements for compliance with COVID-19 restrictions must be emphasized the importance of temporal dimensions and benefits for the future. From the above-mentioned literature, we conclude that psychological reactance arouses from COVID-19 restrictions has an impact on compliance with COVID-19 restrictions when psychological distancing is applied in restaurants during COVID-19; thus, we postulate the following hypothesis.

**H5:** The psychological reactance has a negative relationship with compliance with COVID-19 restrictions.

#### Moderating role of lockdown restrictions

The COVID-19 pandemic came out as a global threat with thousands of deaths and millions of affected people which lead governments to impose some lockdown restrictions to prevent the further spread and contamination of the virus (Lalot et al., 2020). The lockdown restrictions include the closure of educational institutes, including kindergartens, schools, colleges, universities, and businesses like hairdresser shops, cafes, restaurants, salons, boutiques, cinemas, etc. (Lemenager et al., 2021). The travel ban, borders sealing, dismissal of sports events, and other postponement or cancellation of other events like concerts and other social gatherings are also included in these lockdown restrictions (Lemenager et al., 2021). The people being conscious of the severity of the situation and because they trust their authorities, are willing to follow the lockdown restrictions which imposed psychological distancing (Lalot et al., 2020). The lockdown restrictions enhance social, temporal, and spatial distancing. The following are the studies that back up the relations developed.

The lockdown during the COVID-19 pandemic has driven social distancing and made people follow it and also take check on others, whether they follow or not (Sargeant et al., 2021). The research of Foroudi et al. (2021), explained that lockdown restrictions amid the COVID-19 pandemic imposed social distancing, especially in restaurants. The deadly COVID-19 pandemic encouraged governments to impose lockdown which includes social distancing and this distancing leads to making people confide in homes (Lemenager et al., 2021). The governments imposed strict lockdown restrictions to contain the virus and to minimize the adverse effects of the COVID-19 pandemic, the restrictions also include social distancing (Lalot et al., 2020). Based on the mentioned literature we argue that lockdown restrictions increase the social distancing in restaurants, during the COVID-19 pandemic.

Spatial distancing is also called physical distancing. Strict physical distancing is helpful to prevent COVID-19 that's why it is included in the lockdown restrictions (Sheffield et al., 2020). The lockdown restrictions varied in different regions but it mainly consists of physical distancing along with personal safety equipment etc. (Salmon et al., 2021). The physical distancing is proved to be effective to prevent the virus and the deadly effects of COVID-19, which is implemented with the imposition of lockdown (May, 2020). The COVID-19 made governments implement strict policies, to prevent contagion of the virus, these policies contain lockdown restrictions and precautionary measures which all lead to spatial distancing (Bicalho et al., 2021). We infer from previous studies that lockdown restrictions enhance the spatial distancing at restaurants during COVID-19.

COVID-19 encouraged several restrictions and distancing measures which include temporal distancing and these restrictions are imposed through lockdown (Finsterwalder, 2021). The study of Madden et al. (2021), examined the spatio-temporal distancing amid COVID-19 lockdowns and argued how lockdown situations imposed the spatial and temporal distancing. The temporal distancing is implemented during COVID-19 restriction which comprises lockdown restrictions. From the above-mentioned literature, we infer that social, spatial, and temporal distancing which develops psychological distancing in restaurant consumers gets strengthened when lockdown restrictions are perceived to be imposed. Consequently, the following hypothesis is proposed:

**H6:** The lockdown restrictions moderate the relationship between (a) social distancing and psychological reactance, (b) spatial distancing and psychological reactance, and (c) temporal distancing and consumers' psychological reactance.

## Methodology

### Survey instrument

The questionnaire was developed based on prior research. The conceptual framework has seven variables, and the questionnaire is divided into three sections. The first section describes the research, the second section discusses the items used to measure the variables, and the third section discusses the respondents' demographics and controlled variables. For measurement items, we employed a customized Likert scale. The items used to assess perceived social and temporal distancing practices in restaurants during the COVID-19 pandemic were adapted from Kim et al. (2020). We slightly changed this scale to retain the core theme and relevance of measuring social and temporal distance while maintaining the importance of visiting restaurants. The three-item spatial distancing scale was adapted from Cui et al. (2020), which assessed perceived spatial distancing in restaurants during COVID-19. The psychological reactance of food and beverage customers was assessed using the eleven-item (Akhtar et al., 2020) scale, and respondents were asked to anchor their resistance, anger, annoyance, and intrusion. The lockout restriction scale was taken from the work of Foroudi et al. (2021) and operationalized with the key theme of want to visit restaurants in the near future, desire to visit after some time, and realization of the COVID-19 threat to visit restaurants. The freedom to visit restoration is measured using the 4-item scale employed in the study of Milne and Wise (1991) and Smith et al. (2016). The scale for compliance to COVID-19 restrictions is measured using the 12-item scale from Bashir et al. (2021), which has been slightly modified to include avoidance of handshaking, frequent hand washing, hygiene practices, avoiding social meetings, and maintaining distance from others The third section comprises demographics and control variables, such as gender, age, monthly income, and education. We then conducted a pilot study to ensure the measurement's reliability and validity. The Cronbach's alpha value was more than 0.7. Our results show that our survey instrument has good internal consistency.

### Sampling and data collection procedure

The outbreak of the COVID-19 pandemic imposed a threat to the world economy. We looked at the Pakistani food and beverage industry, which is also suffering from COVID-19's effects and getting worse (Javed, 2020). COVID-19 had a large impact on the restaurant industry, and dining activities varied from region to region and country to country, including complete restaurant closures, takeaways, outdoor dining, and re-opening of restaurants with strict adherence to COVID-19 precautionary measures (Elsayed et al., 2021). This pandemic had an effect on the restaurant business in Pakistan, and the COVID-19 crisis decreased the business volume of the restaurants (Burhan et al., 2021). The Pakistani restaurant business is rapidly expanding and is regarded as a significant contributor to GDP, encouraging researchers to investigate restaurant consumers' interests, satisfaction, expectations, and retention (Rahoo and Khan, 2021). That's why we chose the beverages restaurants in Pakistan for this study.

We employed the Kline technique for sample size, which recommended a minimum of 390 (39^*^10) respondents. Therefore, we selected a sample size of 400+ restaurant consumers, which is more than the required sample size. We used Ferber's (1977) sampling technique, and our study sample is relevant to the target population, adequate for analysis, and representative of the population in the subjects being examined. Thus, the self-selected sampling is adequate for generalizing the results (Akhtar et al., 2020).

We collected data from consumers of food and beverage restaurants in Lahore. As a COVID-19 preventative measure, an online survey was used. The online survey method is easy to use and convenient during the pandemic situation like COVID-19 as compared to other offline research methods like experiment research, interviews, observation, etc. (Akhtar et al., 2020). These are the reasons why we selected the online survey method. We employed Google forms to conduct an online survey. The questionnaire began with a brief declaration and promise of confidentiality. Then, thirty-nine closed-ended questions about research variables and four demographics of the respondents were employed. The questionnaire was completed in 15–20 min by the respondents. We received 510 responses from customers of food and beverage restaurants. Out of these, 491 responses were considered final, and 19 were excluded due to extreme values.

## Data analysis

### Demographic results

Table 1 shows that 26.7% of the total respondents were male and 73.3% were female. The age groups of those respondents are in the percentage of 26.7% of 20 years or below, 63.3% of 21 to 30 years, 8.3% of 31–40 years, and finally, 1.7% of 40 years and above. Most of the respondents are aged between 21 and 30 years. 7.7% of the respondents in our study are enjoying a monthly income of fewer than 50,000 rupees. 17.3% of the respondents are getting a monthly income of >100,000 and 7.7% of the respondents are earning more than 500,000 per month. Most respondents earn <50,000 per year. 21.7% of the final respondents have completed intermediate studies, 38.3 percent are graduates, 21.7% have completed master's degrees, and only 18.3% have completed postgraduate studies. Most of the respondents are food and beverage restaurants that are visited once a month, whereas 20% of the respondents are once-a-week visitors, 18.3% are twice-a-week visitors, and 6.7% are daily visitors.

**Table 1:** Respondents' demographics.

**Demographics**	**Frequency**	**Percentage**
**Gender**
Male	131	26.7
Female	360	73.3
**Age**
20 years or below	131	26.7
21–30 years	310	63.3
31–40 years	41	8.3
41 years and above	9	1.7
**Monthly income (Rs)**
<50,000	368	75
>100,000	85	17.3
>500,000	38	7.7
**Education**
Intermediate	106	21.7
Graduation	188	38.3
Master's	106	21.7
Post-graduation	91	18.3
**Frequency of visit to restaurants**
Once a month	270	55
Once a week	98	20
Twice a week	90	18.3
Daily	33	6.7

### Measurement model assessment

This research uses IBM Amos 24 to examine the measurement model, following Anderson and Gerbing (1988) two-step approach. We assessed the constructs' psychometric properties using measurement model fitness. Table 2 shows factor loadings ranging from 0.7 to 0.84, which were above the minimum acceptable value of 0.7. We also excluded CCM4 = 0.086 from the further investigation due to a score below the threshold. The results revealed that all fit indices were acceptable: χ_2_ = 947.723, df = 506, χ_2_/df = 1.873, RMSEA = 0.042 (Hu and Bentler, 1999), AGFI = 0.884 (MacCallum and Hong, 1997), NFI = 0.903, RFI = 0.893, IFI = 0.953, TLI = 0.947 and, CFI = 0.952 were above the threshold value, as suggested by (Hu and Bentler, 1999).

**Table 2:** Factor loadings of confirmatory factor analysis.

**Constructs**	**Items**	**Statements**	**Factor loadings**
Social distance CR = 0.809, AVE = 0.585, α = 0.808	SOD1	I am negatively affected by the COVID-19 pandemic.	0.731
	SOD2	The social distance is essential to me during COVID-19 pandemic.	0.779
	SOD3	The social distance is relevant to me during COVID-19 pandemic.	0.784
Spatial distance CR = 0.770, AVE = 0.528, α = 0.752	SPD1	I can use the spatial distance anywhere during COVID-19 pandemic.	0.762
	SPD2	I can use the spatial distance even while staying at the accommodation during COVID-19 pandemic.	0.700
	SPD3	Using the spatial distance is always suitable at the accommodation during COVID-19 pandemic.	0.719
Temporal distance CR = 0.857, AVE = 0.666, α = 0.856	TPD1	The temporal distance during COVID-19 pandemic will become essential to me in the distant future.	0.830
	TPD2	The temporal distance during COVID-19 pandemic will become relevant to me in the distant future.	0.813
	TPD3	The temporal distance during COVID-19 pandemic will begin to negatively influence me in the distant future.	0.805
Psychological reactance CR = 0.947, AVE = 0.617, α = 0.940	PR1	I consider advice from others to be an intrusion.	0.748
	PR2	It irritates me when someone points out things that are obvious to me.	0.757
	PR3	Advice and recommendations usually induce me to do just the opposite.	0.721
	PR4	Regulations trigger a sense of resistance in me.	0.778
	PR5	I find contradicting others simulating.	0.785
	PR6	When something is prohibited, I usually think, “that's exactly what I am going to do”.	0.755
	PR7	When someone forces me to do something, I feel like doing the opposite.	0.844
	PR8	I resist the attempts of others to influence me.	0.830
	PR9	It makes me angry when another person is held up as a model for me to follow.	0.791
	PR10	I became frustrated when I am unable to make free and independent decisions.	0.795
	PR11	I become angry when my freedom of choice is restricted.	0.828
Freedom to visit restoration CR = 0.810, AVE = 0.588, α = 0.810	FR1	I avoid hand shaking	0.768
	FR2	I usually put a face mask when outside	0.789
	FR3	I stopped attending weddings and social gatherings	0.742
Compliance to COVID-19 measures CR = 0.760, AVE = 0.615, α = 0.792	CCM1	Despite government restriction and lockdown, I wish to go to any restaurants in the near future.	0.719
	CCM2	Despite the government restriction and lockdown, my desire for going to any restaurants in the next 3 months is very strong	0.700
	CCM3	It is dangerous to go to any restaurants because of Coronavirus pandemic and government lockdown policy.	0.746
Lockdown restrictions CR = 0.918, AVE = 0.514, α = 0.910	LR1	I usually go along with others' advice.	0.773
	LR2	I feel it is better to stand up for what I believe than to be silent.	0.725
	LR3	I am very stubborn and set in my ways.	0.782
	LR4	It is very important for me to get along well with the people I work with.	0.799
	LR5	I usually go along with others' advice.	0.775
	LR6	I feel it is better to stand up for what I believe than to be silent.	0.848
	LR7	I am very stubborn and set in my ways.	0.783

Cronbach's alpha measured the internal consistency of the variables' items. According to Hair et al. (2011) the acceptable Cronbach's alpha score is 0.70; all of the values are higher than 0.7. As shown in Table 2, we also calculated the composite reliability (CR). The results were 0.76 and 0.947 above the threshold of 0.7 (Hair et al., 2011). Our results revealed that the average variance extracted (AVE) was 0.5, as advised by Fornell and Larcker (1981). Table 3 shows the AVE results, which range from 0.51 to 0.66. The discriminant validity is examined in two steps. In the first step, the correlations of variables are tested with a threshold value of 0.85 (Kline, 2005), The second step is to confirm the square root of AVE, which should be greater than the correlations of all variables (Fornell and Larcker, 1981), the values for the square root of AVE shown in Table 3. The results of our study confirmed these psychometric properties and model fit indices.

**Table 3:** Discriminant validity.

	**1**	**2**	**3**	**4**	**5**	**6**	**7**
Social distance	**0.765**						
Spatial distance	0.267	**0.727**					
Temporal distance	0.419	0.690	**0.816**				
Psychological reactance	0.554	0.089	0.130	**0.786**			
Freedom to visit restoration	0.137	0.699	0.582	0.196	**0.767**		
Compliance to COVID-19 measures	0.250	0.134	0.093	0.208	0.139	**0.717**	
Lockdown restrictions	0.050	0.038	0.106	0.131	0.135	0.066	**0.784**

*Bold values indicates discriminant validity values*.

### Common method variance

A risk of common method bias (CMB) was present in our study since all the responses came from the same participants. Exploratory factor analysis was used to look into the CMB issue. We performed an unrotated factor solution for all variables. The CMB threshold value is below 50%, which is confirmed by our data, as the results for the first two factors are 27.21 and 13.6%, which total 40.82%. In addition, the variance inflation factor (VIF) results ranged from 1.043 to 2.038, which was below the acceptable threshold of 3 for the collinearity test. Our findings show that common method bias is not a problem for our investigation.

### Structural model assessment and hypotheses results

We employed structural equation modeling (SEM) to investigate the presented hypotheses and model fitness. We used a variety of goodness of fit indices to verify the model's fitness. The results revealed the following good fit indices: [χ_2_ = 850.792, df = 316, χ_2_ /df = 2.692, GFI = 0.893, AGFI = 0.872, TLI = 0.916, CFI = 0.925, RFI = 0.873, NFI = 0.886, IFI = 0.925 (Hair et al., 2011), PGFI = 0.746, PCFI = 0.832, PNFI = 0.797, RMSEA = 0.059] (MacCallum and Hong, 1997).

**Figure 2:**
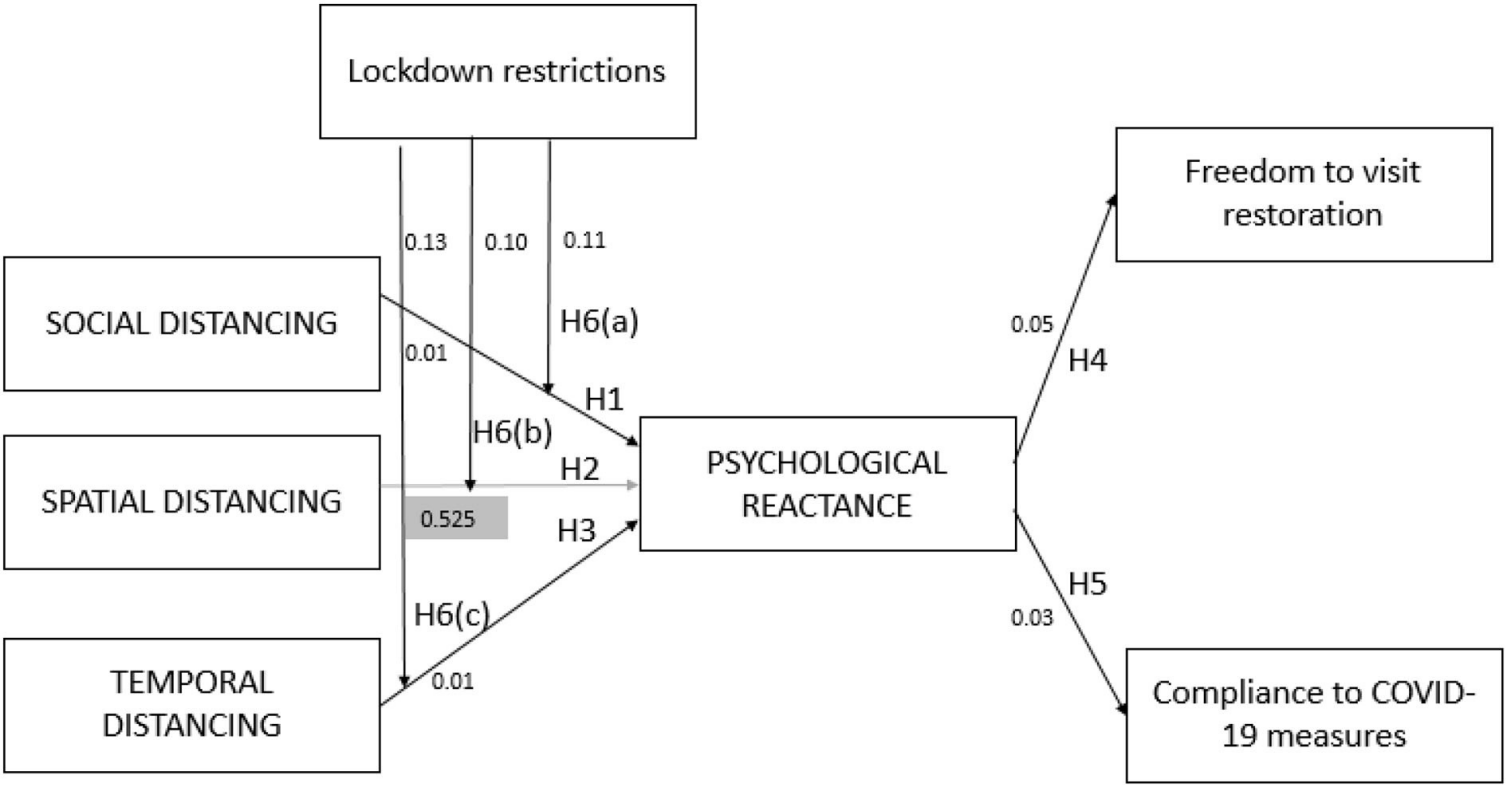
Validation of hypothetical relationships.

We used structural equation modeling (SEM) to test the hypotheses (see Table 4). The results are also represented in the Figure 2. The results of structural relationships showed that social distance had a significant positive influence (*H*1_*SOD*→*PR*_ = 0.642, *t* = 6.544, *p* < 0.01) on consumers' psychological reactance, which supports and confirms our first hypotheses. The spatial distance had a significant positive influence (*H*2_*SPD*→*PR*_ = 0.059, *t* = 0.635, *p* < 0.525) on consumers' psychological reactance, which also supports our second hypothesis. We found a significant positive relationship between temporal distance and consumer psychological reactance (*H*3_*TPD*→*PR*_ = 0.136, *t* = 3.372, *p* < 0.01), supporting H3. The result of the fourth hypothesis showed that consumer psychological reactance had a positive effect on freedom to visit restoration (*H*4_*PR*→*FR*_ = 0.144, *t* = 2.79, *p* = 0.05), thus H4 is supported. The fifth hypothesis is also supported as psychological reactance has a significant positive effect on compliance with COVID-19 measures (*H*5_*PR*→*CCM*_ = 0.159, *t* = 2.963, *p* = 0.03).

**Table 4:** Confirmation of proposed hypotheses.

**Paths**	**β**	***t*-statistics**	**Path coefficients**	**Relationship**
*H*1_*SOD*→*PR*_	0.642	6.544	^***^	Supported
*H*2_*SPD*→*PR*_	0.059	00.635	0.525	Unsupported
*H*3_*TPD*→*PR*_	0.136	3.372	^***^	Supported
*H*4_*PR*→*FR*_	0.144	2.790	0.005	Supported
*H*5_*PR*→*CCM*_	0.159	2.963	0.003	Supported

*** p = 0.001, **p = 0.01, *p = 0.05.

In this study, the predicting variance (*R*^2^) for dependent variables is examined. The value predicts the effects of all independent variables on a dependent variable, which causes variation in the dependent variable. The threshold value of *R*^2^ of each dependent variable described by the independent variables is a minimum of 10%, as recommended by Falk and Miller (1992). The results demonstrated a 51% variance in consumers' psychological reactance, a 21% variance in freedom to visit restoration and a 25% variance in compliance with COVID-19 measures. These results show that *R*^2^ values are greater than the threshold value of 10%. The effect sizes (*f*^2^) are also calculated to check the essential effects of the model for this study. Effect size (*f*^2^) can be defined as the degree of representation of the observed phenomenon in the population studied (Cohen, 1988). Cohen (1988) classification identifies the effect sizes (*f*^2^), which is basically the variance expounded by all other independent variables. The classification of effect sizes is 0.02 as small, 0.15 for medium effect, and 0.35 for large effect. The effect size (*f*^2^) for consumers' psychological reactance is 1.045, which is a high impact size; the effect size (*f*^2^) for freedom to visit restoration is 0.0256, and the effect size (*f*^2^) for compliance with COVID-19 measures is 0.0215, which are the small effect size.

### Moderation results

The moderating effects of lockdown restrictions were analyzed separately for each of the three antecedents. The effect of lockdown restrictions on the influence of social distance on psychological reactance was found to be significant (*H*6_(*a*)_−β_*LR*×*SOD*→*PR*_ = 0.1357, *t* = 3.9567, *p* = 0.0001, *CI* = 0.0683, 0.2031), which supports H6(a). Furthermore, the study found that lockout restrictions had a significant moderating influence on the relationship between spatial distance and consumer psychological reactance (*H*6_(*b*)_−β_*LR*×*SPD*→*PR*_ = 0.1041, *t* = 3.0520, *p* = 0.0024, *CI* = 0.0371, 0.1712), is supported H6(b). We also confirmed the moderating effect of lockdown restrictions on the effect of temporal distance on consumers' psychological reactance (*H*6_(*c*)_−β_*LR*×*TPD*→*PR*_ = 0.1112, *t* = 3.3011, *p* = 0.001, *CI* = 0.045, 0.1775), is supported hypothesis 6(c). Table 5 also depicts the results in a summarized manner. These findings confirm the moderating effect of lockdown restrictions on the relationships between social, spatial, and temporal distance and psychological reactance.

**Table 5:** Direct and moderating effects.

**Paths**	**Path coefficients**	***t*-statistics**	**Bias corrected CI**	**Relationship**
*H*6_(*a*)_−β_*LR*×*SOD*→*PR*_	0.1357	3.9567	(0.0683, 0.2031)	Supported
*H*6_(*b*)_−β_*LR*×*SPD*→*PR*_	0.1041	3.0520	(0.0371, 0.1712)	Supported
*H*6_(*c*)_−β_*LR*×*TPD*→*PR*_	0.1112	3.3011	(0.0450, 0.1775)	Supported

## Discussions and implications

The COVID-19 pandemic has altered how individuals connect and travel outside of their homes. It has created a new normal in the world, and everyone must follow the WHO's preventive measures to avoid the devastating repercussions of this pandemic. In response, we investigated how psychological distance causes consumer reactance, which eventually results in freedom to visit restoration and influences compliance with COVID-19 restrictions. With the expansion of consumer psychological reactance (Akhtar et al., 2020; DeFranza et al., 2021; Hajek and Häfner, 2021), we explored how it influences customer compliance with COVID-19 measures at food and beverage restaurants. During the COVID-19 pandemic, the authors looked at how food and beverage restaurant customers perceived their freedom of interaction and mobility. We provide answers to the questions and add to the body of knowledge in psychology and consumer behavior about how customers perceive social, spatial, and temporal distance as threats to perceived freedom.

Initially, the result of our study unveils that, perceived social and temporal distancing measures at food and beverage restaurants make the consumers feel uncomfortable and angry. This feeling of discomfort and anger results in the development of psychological reactance which supports the findings of Cerbara et al. (2020) and Williams (2020), enlightening that psychological distancing results in the development of psychological reactance. The results of our study proved the relationship of spatial distance with consumers' psychological reactance to be insignificant. The people feel more negative emotions because of the social isolation rather than spatial distancing and physical distance can help people to stay safe from COVID-19 (Abel and Mcqueen, 2020). Physical distancing is prescribed to be an effective preventive measure for the COVID-19 virus which people actually adopt out of fear of getting sick or dying (Fini et al., 2020). Thus, with the insignificant results of this relationship, it is proved that restaurant consumers are willing to adopt spatial distance measures during the COVID-19 pandemic. Afterward, our findings confirm that consumer experiencing such reactance tries to restore their perceived freedom to visit, which is also claimed by the studies (Heilman and Toffler, 1976; Moore and Fitzsimons, 2014; Smith et al., 2016; Bessarabova et al., 2017; Zhang, 2020). In addition to these findings, our results confirmed that this consumer reactance affects the consumers' compliance with COVID-19 measures at food and beverage restaurants. This finding was also previously confirmed in the previous literature (Hajek and Häfner, 2021; Mohammed Salih and Fadlalmola, 2021). The results of our study also conclude that lockdown restrictions amid COVID-19 also strengthen the psychological distancing at food and beverage restaurants.

### Theoretical implications

Initially, the result of our study unveils that, perceived social and temporal distancing measures at food and beverage restaurants make the consumers feel uncomfortable and angry. This feeling of discomfort and anger results in the development of psychological reactance which supports the findings of Cerbara et al. (2020) and Williams (2020), which enlightened that psychological distancing results in the development of psychological reactance. This study has contributed to the literature on psychological distancing by examining the perceived social, spatial, and temporal distancing in the context of food and beverage restaurants during COVID-19. The examination of psychological distancing leading to reactance in consumers of food and beverage restaurants which motivates them for freedom to visit restoration and affects the compliance to COVID-19 restrictions also contributes to the literature on construal level theory (CLT). Afterward, our findings confirm that consumer experiencing such reactance tries to restore their perceived freedom to visit, which is also claimed by the studies (Heilman and Toffler, 1976; Moore and Fitzsimons, 2014; Smith et al., 2016; Bessarabova et al., 2017; Zhang, 2020). This finding critically explains that social distancing creates consumer reactance more than temporal distancing as the results (*H*1_*SOD*→*PR*_ = 0.642, *t*= 6.544, *p* < 0.01). The examination of freedom to visit restoration in the food and beverage restaurants, encouraged by reactance arousal because of perceived psychological distancing extends the literature on the freedom to visit restoration and confirmed some new predictors for freedom to visit restoration.

The results of our study proved the relationship of spatial distance with consumers' psychological reactance to be insignificant. The spatial distance does not result in a momentous psychological reactance. The people feel more negative emotions because of the social isolation rather than spatial distancing and physical distance can help people to stay safe from COVID-19 (Abel and Mcqueen, 2020). Physical distancing is prescribed to be an effective preventive measure for the COVID-19 virus which people actually adopt out of fear of getting sick or dying (Fini et al., 2020). Thus, with the insignificant results of this relationship, it is proved that restaurant consumers are willing to adopt spatial distance measures during the COVID-19 pandemic. The restaurants can apply the spatial distance without the fear of consumer reactance and make the customers aware of the importance of staying at a 6-feet distance. This finding relates to the research of Abel and Mcqueen (2020), which exclaims that spatial distance can be useful to prevent COVID-19 disease and people can follow this preventive measure themselves, to avoid the disease. From this finding, our study extends the literature on COVID-19 as well.

In addition to these findings, our results exclaimed that this consumer reactance affects the consumers' compliance with COVID-19 measures at food and beverage restaurants. This finding was also previously confirmed in the previous literature (Hajek and Häfner, 2021; Mohammed Salih and Fadlalmola, 2021), that the reactance developed by distancing measures affect the compliance to COVID-19 measures. The results of our study also conclude that lockdown restrictions amid COVID-19 also strengthen the psychological distancing at food and beverage restaurants. In conclusion, our findings confirm that the perceived psychological distancing at food and beverage restaurants amid lockdown restrictions develops psychological reactance in the consumers which motivate consumers for freedom to visit restoration. This consumer reactance also affects the consumers' compliance with COVID-19 measures and food and beverage have to increase their compliance with COVID-19 restrictions amid the government guidelines and the COVID-19 pandemic threat. The lockdown restrictions proved to be a good moderator for the psychological distancing and consumer reactance as the results confirm. These results also align with the findings of Foroudi et al. (2021), who state that lockdown restrictions enhance distancing measures and develop negative cognitions and anger in the people, which give rise to reactance. But there is insufficient research on lockdown restrictions as moderators which we have examined and confirmed. That's how research also extended the COVID-19 literature by confirming lockdown restrictions as a moderator for the relationship between perceived psychological distancing and consumer reactance, which is a major contribution to the literature on lockdown restrictions.

Likewise, major findings highlight the pre-visit context of food and beverage consumers and find that the consumers develop reactance toward the perceived psychological distancing in the food and beverage restaurants. The food and beverage restaurant industry already faced a lot of challenges during the lockdown and COVID-19 pandemic, that's why they really some effective policies to combat the further challenges which have been arising in the new normal. The food and beverage restaurants also need maximum compliance to COVID-19 measures by their customers which is also affected by the reactance developed, the restaurants must devise their policies to reduce the negative influence of perceived psychological distancing to minimize the reactance and increase the compliance to COVID-19 measures. The restaurants must need to do this to save themselves from penalties and closures. This prior mentioned finding encompasses the earlier conclusions of the studies (Lakshmi and Shareena, 2020; Burhan et al., 2021), particularly in the Pakistani context (Javed, 2020). The study has also contributed to the literature on compliance with COVID-19 measures by examining it in the context of the food and beverage restaurant industry. Compliance with COVID-19 measures is an important topic amid the pandemic as discussed in the study (Bashir et al., 2021), and discussing how the consumer reactance developed by perceived psychological distancing affects the compliance to COVID-19 measures, is a good contribution to the existing body of knowledge.

### Practical implications

This study reveals several implications for the food and beverage restaurant industry. First, customers of food and beverage restaurants are forced to keep a social distance from the restaurants, which generates reactance in the consumers. Our findings show that customers of food and beverage restaurants perceive social distancing as a threat to their freedom of interaction in restaurants. When considering social distancing, some customers choose to eat at home with freedom (Kim et al., 2021), and restaurants must reduce consumers' concern about perceived social distancing. While social distancing is unavoidable, food and beverage restaurants implement additional creative practices to reduce social distancing measures within the restaurants and to mitigate consumers' negative perceptions of social distancing.

Second, we discover that consumers of food and beverages feel uncomfortable when they consider the spatial distancing present in restaurants. Restaurants should adopt innovative dining techniques, such as two-person tables or chair placement based on the necessary spatial distance. The influence of spatial distancing in the mind of consumers will be reduced by innovative dining. One example is the use of blow-up dolls to fill empty seats at the open-heart restaurant in Greenville country. During COVID-19, ETEN in Amsterdam has taken a highly innovative approach to dine by installing separate greenhouses for consumers to have dinner near the Oosterdok harbor.

Third, it has been established that temporal distance causes reactance in food and beverage consumers during COVID-19. COVID-19 restrictions limit consumers' freedom to visit restaurants at any time. They develop a sense of unease and desire to restore perceived freedom, which often affects their restaurant visits. Restaurants can create attractive rules to follow the government's COVID-19 schedules. They can offer extra incentives to dine at certain restaurants, and then offer deliveries, takeaways, or both.

Fourth, the restaurants need their customers to follow COVID-19 guidelines, or they could be fined or even banned from serving food. This study indicates that social and temporal distancing in restaurants affects compliance with COVID-19 measures. Restaurants may offer discounts or other appealing promotions to demonstrate their commitment to strict compliance with COVID-19 guidelines. The lockdown impacted the restaurant industry significantly, which had adverse repercussions during the deadly COVID-19 outbreak. By using COVID-19 prevention measures, the restaurant business can help reduce the number of infected people, reducing the need for lockdowns.

### Limitations and future directions

This study also contains several limitations, which provide directions for future research. First, this study investigated the consumer reactance to psychological distancing at food and beverage establishments, which ultimately leads to freedom to visit restoration and influences compliance with COVID-19 restrictions. Future research can examine this conceptual model in relation to the trait and state reactance elements of psychological reactance. Second, we excluded the fourth dimension of psychological distancing (i.e., hypothetical distancing), and future research can incorporate hypothetical research to investigate this conceptual framework. Third, this conceptual framework is founded on perceived distancing and its reactance. The authors of this work proposed examining this model with real-time distance measures, i.e., the distancing measures that could be used when customers visit restaurants. Fourth, we looked at two different outcomes (i.e., freedom to visit restoration and compliance with COVID-19 measures). Future researchers might also investigate whether the consumer's psychological distancing affects the consumers' intention to revisit the restaurants or not. Lastly, we investigated the boundary condition effects of lockdown restrictions. However, the lockdown restriction can be utilized to mediate the interaction between customer psychological distancing and consumer reactance.

## Data availability statement

The raw data supporting the conclusions of this article will be made available by the authors, without undue reservation.

## Author contributions

XC developed the idea and design the conceptual framework. HI written the initial draft, refine research ideas, collected data, and completed the discussion of the research implications. YacD and YahD edited initial draft an revise logical flow. YacD and YahD revised the manuscript and analysis the data and interpretation. All authors contributed to the article and approved the submitted version.

## Funding

We are grateful for the financial support provided by the National Philosophy and Social Science Foundation (Grant No. 19BYY016), the Planning Funds of the Beijing Key Project of Philosophy and Social Science (Grant No. 16YYA005), and the Project Funded by the Ministry of Education (Grant No. 19JHQ035).

## Conflict of interest

YD was employed by the company China Renaissance. The remaining authors declare that the research was conducted in the absence of any commercial or financial relationships that could be construed as a potential conflict of interest.

## Publisher's note

All claims expressed in this article are solely those of the authors and do not necessarily represent those of their affiliated organizations, or those of the publisher, the editors and the reviewers. Any product that may be evaluated in this article, or claim that may be made by its manufacturer, is not guaranteed or endorsed by the publisher.
